# Single Feature Polymorphism Discovery in Rice

**DOI:** 10.1371/journal.pone.0000284

**Published:** 2007-03-14

**Authors:** Rajesh Kumar, Jing Qiu, Trupti Joshi, Babu Valliyodan, Dong Xu, Henry T. Nguyen

**Affiliations:** 1 Division of Plant Sciences, University of Missouri-Columbia, Columbia, Missouri, United States of America; 2 Department of Statistics, University of Missouri-Columbia, Columbia, Missouri, United States of America; 3 Computer Science Department and Christopher S. Bond Life Sciences Center, University of Missouri-Columbia, Columbia, Missouri, United States of America; University of Chicago, United States of America

## Abstract

The discovery of nucleotide diversity captured as single feature polymorphism (SFP) by using the expression array is a high-throughput and effective method in detecting genome-wide polymorphism. The efficacy of such method was tested in rice, and the results presented in the paper indicate high sensitivity in predicting SFP. The sensitivity of polymorphism detection was further demonstrated by the fact that no biasness was observed in detecting SFP with either single or multiple nucleotide polymorphisms. The high density SFP data that can be generated quite effectively by the current method has promise for high resolution genetic mapping studies, as physical location of features are well-defined on rice genome.

## Introduction

The publicly available genome sequence information of rice [1] opens a great opportunity for facilitating and integrating various genomics studies, for example, isolation of genetic determinants associated with traits of economic importance. Such discovery has not only promises to complement the molecular breeding efforts but also speeding up the process of crop improvements in general by incorporating useful genes into agronomically suitable varieties through genetic engineering. Additionally, information obtained with rice can also be translated into other crops as well because of high conservation of synteny observed among related cereal crop species [2].

The nucleotide diversity across a genome is the source of most of the phenotypic variation. Such DNA polymorphism is the basis for development of molecular markers, an indispensable tool in genetic mapping studies. In general, the high resolution fine mapping of genes is often limited by lack of sufficient number of polymorphic molecular markers. This problem is compounded with traits controlled by multi-genes because in several such studies QTL locus can't be resolved to a workable resolution that could be feasible for predicting the candidate gene(s) associated with traits of interests. The sequence comparison of Nipponbare and 93-11 genome has shown high degree of polymorphisms ranging from a SNP/∼300 bp to an indel/kbp [3]–[4] that can potentially be exploited as molecular markers between these two genetically diverged sub-species. In genetic mapping studies PCR-based SSR and CAPS markers are used routinely and generation of such molecular markers becomes easier if one of the two parents of mapping population has sequence information available. Many varieties of rice endowed with different traits of economic interests are grown worldwide. In situations where both parents of mapping population lack sequence information and are in similar genetic background often requires multiple steps in the process of identification and generation of such PCR based molecular markers.

There are several ways through which polymorphisms are identified across the genome. The most direct method is to re-sequence the PCR amplicons, the DNA fragments containing alleles from the inbred parents. Similarly EST sequences also provide the direct way to compare the sequence and provide useful information about polymorphism. These methods although straight forward in approach but are quite labor intensive and lack high-throughput. The high density oligonucleotide expression arrays, designed for transcript profiling, have been used successfully as an effective tool for DNA genotyping to measure numerous polymorphic loci in yeast [5] and Arabidopsis [6]. Application of such DNA-based technique in complex genome like barley however were not as sensitive as it was reported in Arabidopsis but when RNA was used as surrogate for DNA the efficiency in predicting polymorphism increased significantly [7]–[8]. The basis of genome wide polymorphism discovery by the above approach is dependent on the principle that a sequence which is perfect match to a feature/probe sequence present on gene-chip or array may hybridize with greater affinity than one with a mismatch sequence. The polymorphism of the two sequences, originating from two different varieties or genotype, results in differential hybridization intensity and this property associated with sequence characteristics functions as a molecular marker popularly known as **s**ingle **f**eature **p**olymorphism (SFP) [5]–[6].

The objective of present study was to test the efficacy of such DNA-based gene-chip approach to identify polymorphism in rice. Here we demonstrate that hybridization of probes, generated from labeling of g-DNA, to rice whole genome expression array (Affymetrix) is quite sensitive in predicting SFP *a priori* of their sequence information. The rice varieties used in this study were Cypress (CP), LaGrue (LG) and RT0034 (RT); first two belong to japonica [9] whereas RT0034 to indica background [10] respectively. These three varieties constitutes parents of two population developed by RiceCAP [10] for mapping QTLs/genes associated with economically important milling yield trait. Milling yield is a complex quantitative trait [11] and considered as products of numerous loci with varying degrees of effect upon the observed phenotypes. Since SFPs can reliably be predicted in rice, the methods presented in the paper can be applied to any rice varieties irrespective of their sequence information for not only polymorphism discovery but also as a tool for functional genotyping of natural varieties.

## Results

### Hybridization and data quality

**Figure 1:**
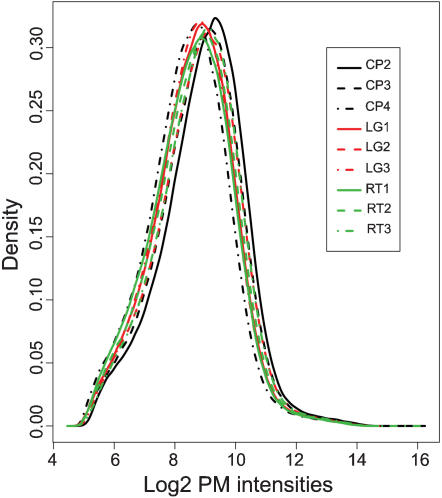
Density plots for the raw PM probes intensity data. The data are in log2 scale and the biological replicate arrays for rice varieties CP, LG and RT are shown in black, red and green color respectively. CP = Cypress, LG = LaGrue, RT = RT0034 and PM = perfect match.

**Figure 2:**
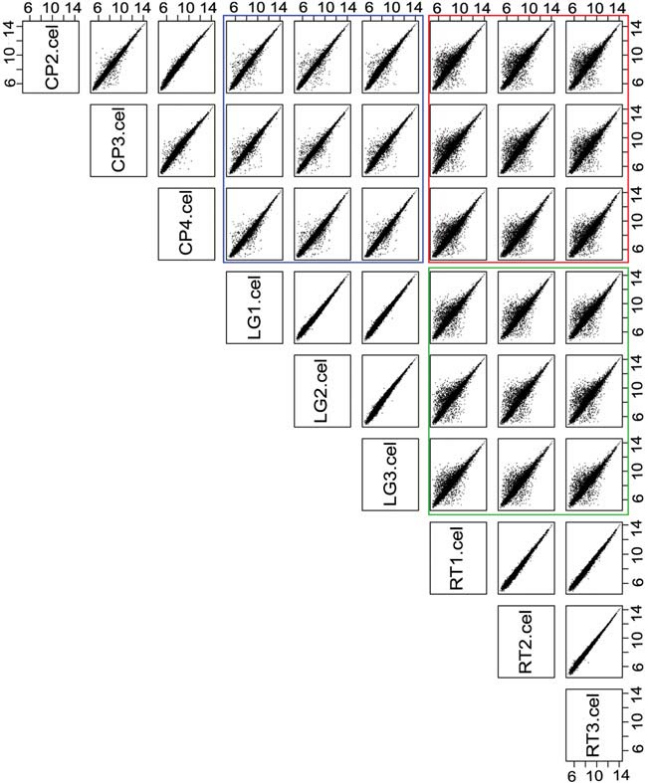
Pair-wise scatter plots for the raw PM probes intensity data across all arrays. The intersection of 12000 randomly chosen features/probes data for CP and LG is boxed in blue, for CP and RT in red and for LG and RT in green respectively. The biological replicates of each variety are highly correlated as features are falling along diagonal line. CP = Cypress, LG = LaGrue, RT = RT0034 and PM = perfect match.

The biotin labeled probes generated from labeling of g-DNA was hybridized to Affymetrix rice expression array (see experimental procedure). Following hybridization the preliminary data quality was assessed from GCOS1.3 software (Affymetrix) generated expression report according to guidelines (see Affymetrix manual) set for such experiments. The average background, noise (RawQ) and the call rate was comparable among all the three rice varieties viz. Cypress (CP), LaGrue (LG) and RT0034 (RT) and also among their biological replicates (data not shown). However to get better assessment of data quality the raw intensity data of only perfect match (PM) probes/features of rice varieties viz. CP, LG and RT were log2 transformed and studied by density plots (Figure 1) and pair-wise scatter plots (Figure 2) respectively. The results obtained from density plot indicated no major deviations as replicates of rice varieties were correlated to each other. For scatter plot study 12000 randomly chosen features were plotted against each other for all pair-wise combinations (Figure 2) as suggested in Borevitz's methods paper [12]. No major variation was observed among biological replicates of each variety as most of the features were falling along the diagonal. The features falling above or below diagonal lines indicate their differential hybridization intensity and thus qualify for SFPs. The number of such features showing differential hybridization in CP&LG (blue box) was much less than those in CP&RT (red box) or LG&RT (green box) pairs respectively as one would expect between varieties of same than to different genetic background.

### SFP prediction

The background corrected and quantile-normalized log2 intensity values of all PM features of triplicate data of each rice variety were subjected to SFPs call by using the siggene package (www.boiconductor.org) and SAM procedure in R language software essentially as described [7], [12]–[13]. SAM procedure allows the users to choose the delta value, a threshold for the SAM d-statistics, so as to get a balanced number of significant genes or SFPs as in present study with a tolerable FDR, which is estimated by permutation [14]. In this paper, the FDR that is determined by permutation according to the SAM procedure is referred to the *estimated FDR* and the FDR that is determined after sequencing is referred to the *observed FDR*. The SFPs called at different threshold (delta) in the three datasets viz. CP&LG (a), CP&RT (b) and LG&RT (c) is presented in Table 1. Since the estimated FDR was stable for a wide range of delta values (data not shown), meaning a larger number of significant SFP would imply a larger number of false positives although the proportion of false positive doesn't change; we elected to choose the largest number of SFPs at the given stable estimated FDR for their verification by sequence analyses. Based on above consideration we selected 5376 SFPs that were called with an estimated 9.5% FDR in CP&LG pair (Table 1a) and 25325 SFPs for CP&RT pair at an estimated 9% FDR (Table 1b).

**Table 1:** Number of gene-chip predicted SFPs called at different threshold (delta) in datasets.

(a) CP&LG					(b) CP&RT					(c) LG&RT				
Delta	p0	FALSE	Called	FDR	Delta	p0	FALSE	Called	FDR	Delta	p0	FALSE	Called	FDR
1	0.95	3128.7	13668	0.217	1	0.95	16942.4	110346	0.146	1	0.95	12349	92926	0.126
2	0.95	1134.4	9093	0.119	2	0.95	6457.15	61055	0.100	1.2	0.95	9652.9	80935	0.113
3	0.95	719.8	6865	0.100	3	0.95	4558.5	46306	0.094	1.4	0.95	8131.8	72951	0.106
4	0.95	537.7	5376	0.095	4	0.95	3580.15	37137	0.092	1.6	0.95	7165.1	67299	0.101
5	0.95	423.55	4274	0.094	5	0.95	2905.7	30322	0.091	1.8	0.95	6493.4	62756	0.098
6	0.95	344.85	3488	0.094	6	0.95	2404.95	25325	0.090	2	0.95	5983.9	58918	0.096

The data were analyzed as described [7]. (p0 = the prior probability of the proportion of SFP in the null datasets; Called = the number of SFP at each threshold; False = the number of SFP in the mean permuted dataset; FDR = false discovery rate; CP = Cypress; LG = LaGrue and RT = RT0034.

**Figure 3:**
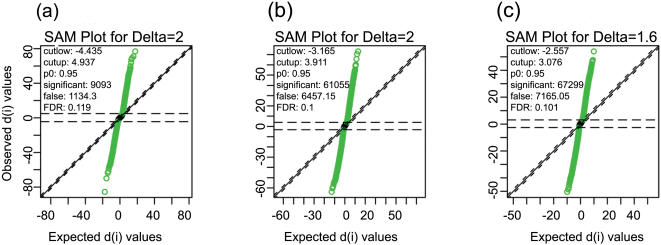
SAM plot of normalized data for CP&LG (a) and CP&RT (b) and LG&RT(c) pairs. Observed d-statistics (y-axis) is plotted against the expected d-statistics (x-axis) as determined by permutations and SFPs exceeding the threshold are shown in green. The sign (+/−) with SFPs indicates direction of polymorphism. In (a) the (-) sign (i.e. CP-SFP) indicates polymorphism in LG (i.e. CP>LG) and (+) sign (i.e. LG-SFP) polymorphism in CP (i.e. LG>CP); in (b) the (-) sign (i.e. CP-SFP) signifies polymorphism in RT(i.e. CP>RT) and the (+) sign (i.e. RT-SFP) polymorphism in CP(i.e. RT>CP) and in (c) the (-) sign (i.e. LG-SFP) indicates polymorphism in RT (i.e. LG>RT) and (+) sign (i.e. RT-SFP) polymorphism in LG (i.e. RT>LG). CP = Cypress, LG = LaGrue and RT = RT0034.

The distribution of gene-chip predicted SFPs (at ≥10% estimated FDR) among polymorphic probesets in the three datasets is shown in Table 2. The observation of ∼6-7xs polymorphism in CP&RT or LG&RT datasets compared to CP&LG in the present study is therefore in agreement with significantly higher genetic divergence between japonica and indica variety of rice [3]–[4], [15] than those observed between varieties of similar genetic background [16]. The SAM plot of normalized data of all the PM probes on array for all the three datasets is shown in Figure 3. The probes exceeding the threshold, shown in green color, signify SFPs, sign (+/−) associated with SFP indicates direction of polymorphism and the values as its SAM d-stat value.

**Table 2:** Distribution of gene-chip predicted SFPs among polymorphic probesets.

Datasets	Number of SFPs per probeset										
	1	2	3	4	5	6	7	8	9	10	11
**CP&LG**											
(−)SFP-PS	1255	439	213	132	106	69	64	51	53	36	17
(+)SFP-PS	720	218	103	50	45	33	25	21	19	18	17
**(total)**	**1975**	**657**	**316**	**182**	**151**	**102**	**89**	**72**	**72**	**54**	**34**
**CP&RT**											
(−)SFP-PS	6994	3377	1774	1068	674	529	430	421	325	311	264
(+)SFP-PS	5252	1187	454	266	168	129	104	98	99	98	73
**(total)**	**12246**	**4564**	**2228**	**1334**	**842**	**658**	**534**	**519**	**424**	**409**	**337**
**LG&RT**											
(−)SFP-PS	7182	3424	1864	1054	732	549	406	391	368	330	304
(+)SFP-PS	5809	1456	648	388	259	181	142	142	132	140	115
**(total)**	**12991**	**4880**	**2512**	**1442**	**991**	**730**	**548**	**533**	**500**	**470**	**419**

The probesets (PS) and SFPs data have been taken from estimated FDR (delta) of 11.9% (delta = 2) in CP&LG, 10% (delta = 2) in CP&RT and 10% (delta = 1.6) in LG&RT respectively. The sign (+/−) indicates direction of polymorphism as explained in Figure 3 legend. CP = Cypress, LG = LaGrue and RT = RT0034.

### SFP verification

To test the sensitivity of gene chip predicted SFPs data we verified them by sequence comparison. To the best of our knowledge no sequence information was available for rice varieties Cypress (CP), LaGrue (LG) and RT0034 (RT) and due to this limitation fragments flanking the SFPs were amplified from their respective genome (see experimental procedure) and their sequences was compared to verify polymorphism, if any. To simplify and maintain uniformity we validated SFPs whose corresponding 25mer probe/feature sequence had unique location, 100% identity and distributed randomly on all 12 chromosomes (supplementary Table S1 & S2) of Nipponbare genome (TIGRv3) sequence available during the course of experiment. We generated sequence information of altogether 186 probes having unique location for CP&LG pair. The 104 probes were having known sequence polymorphism and 77 were predicted correctly at 9.5% estimated FDR by SAM procedure suggesting 74% sensitivity of SFP detection (Table 3a). Similarly for CP&RT pair we generated sequence information of 603 probes having unique location and among the 245 probes having known sequence polymorphism 180 were predicted correctly at 9% estimated FDR by SAM procedure suggesting 73% sensitivity (Table 3b). The direction of polymorphism was correct in all of the sequence verified SFPs in both the pairs/datasets except four in CP&RT pair. Such rare reversal of polymorphism has also been observed with SFP study in barley [7].

**Table 3:** Verification of gene-chip predicted SFPs by sequence information.

(a) SFP in CP&LG			
Sequence information		Gene-chip	
		**5376 SFP(9.5% FDR)**	**9093 SFP (11.9% FDR)**
Total probe sequence	186	78	85
Polymorphism	104	77	82
Non-polymorphism	82	1	3
**SFP detection sensitivity**		74%	79%
**FDR after sequencing**		1%	3%
**(b) SFP in CP&RT**			
**Sequence information**		**Gene-chip**	
		25325 SFP(9% FDR)	61055 SFP(10% FDR)
Total probe sequence	603	204	257
Polymorphism	245	180	207
Non-polymorphism	358	24	50
**SFP detection sensitivity**		73%	84%
**FDR after sequencing**		12%	20%

CP = Cypress, LG = LaGrue and RT = RT0034.

Since a significant proportion (∼25%) of probes having known sequence variation in both the data set escaped from being predicted as SFP at the given analyzed FDR, we explored the possibility of finding such SFPs at less stringent estimated FDR. In CP&LG dataset at 11.9% estimated FDR the number of predicted SFP nearly doubled to 9093 compared to those predicted at 9.5% estimated FDR (Table 1a). From the available sequence information, we found that out of 27 SFPs that escaped detection at 9.5% FDR, five were predicted correctly at 11.9% FDR and thus increasing the sensitivity further to 79% (Table 3a). Similarly at estimated FDR of 10% the number of SFPs predicted in CP&RT dataset nearly doubled to 61055 (Table 1b) including 35730 unique SFPs. Based on available probe/feature sequence information, we verified sequences of 257 SFPs predicted at 10% FDR. Among the 245 probes having known sequence polymorphism, 207 were predicted correctly increasing the sensitivity to 84% (Table 3b) and the 27 additional SFP were those that escaped detection at 9% estimated FDR. Although by lowering the stringency of estimated FDR, the sensitivity of SFP detection was increased substantially in CP&RT dataset but simultaneously the observed FDR determined after sequencing also doubled (20%) compared to the estimated value by permutation (Table 3b).

The higher the value of d-stat of SFP, the greater is the likelihood of being predicted true [7]. In CP&RT dataset, among the 204 sequence verified SFPs, 24 turned out to be false positive indicating marginally higher FDR (11.7%) compared to the estimated value (9%) by permutation. Since we couldn't find SFPs with higher d-stat value being false positive in both the datasets and all the false positives in CP&RT dataset had d-stat values close to permuted cut-off value (data not shown) suggesting that most false positives, if not all, would be among the SFPs that has d-stat value close to permuted cut-off level. In CP&LG dataset most of the SFPs, which were verified by sequencing, had higher d-stat value (data not shown) and that could possibly explain why we couldn't find number of false positives determined after sequencing comparable to that of permuted value.

In genetic mapping studies theoretically a probeset/gene having either one or multiple SFP will provide the same information if such probeset/gene having their defined position on genome is being exploited as molecular marker. According to result presented in Table 2, the number of SFPs per probeset and the number of polymorphic probesets were quite variable in all the three datasets. Since ∼55% of the total polymorphic probesets were having only one SFP each in all three datasets, we estimated how many of such polymorphic probesets were true positive. Among the sequence verified SFP predicted at 9.5% estimated FDR in CP&LG dataset (Table 3a), 11 probesets had one SFP each and none escaped detection (data not shown). On the other hand among the sequence validated SFPs predicted at 9% estimated FDR in CP&RT dataset (Table 3b), 47 probesets were having one SFP each and all were true positive except five and the d-stat values of SFPs associated with such false positive probesets were close to permuted cut-off level (data not shown). The absence of such false positive probesets in CP&LG dataset might be due to either higher d-stat value of sequence verified SFPs as mentioned earlier and/or less number of such probesets were analyzed compared to CP&RT dataset.

### Nature of polymorphism and detection sensitivity

SNPs (single nucleotide polymorphisms) are the most frequent form of polymorphism observed in any organism. Given their wide application in genetic fine mapping studies, it was of interest to analyze how many of probes/features having known SNP was detected by this method. From available sequence information in CP&LG dataset 46 probes were having SNP but only 29 were predicted correctly indicating 63% detection efficiency (Table 4a). On the other hand in CP&RT dataset 144 probes were having SNP and 108 were predicted correctly suggesting 75% efficiency (Table 4b). The less detection efficiency of SFP having SNP in CP&LG dataset could be due to less number of such SFPs were analyzed compared to SFPs with multiple nucleotide polymorphisms (NP). Based on similar nature of previous study [8] SFPs containing SNP were classified into two category viz. SNP residing either at margin (flanking1–5 bases) or in the middle (6–20 bases) of 25mer probe/feature (Table 4). Although the number of sequence verified SFPs having SNP at the flanking 1–5 bases were comparatively less than those present in the middle of features, the present study reconfirms the poor detection of SFPs having SNP situated in the flanking 1–5 bases than those present in middle of features/probes [7]–[8]. The above observation is better explained at least by CP&RT dataset where both the number of SFPs with SNP and also ratio of SFPs with SNP to SFPs with multiple NP were comparatively higher than that of CP&LG dataset. We further compared the detection sensitivity of SFPs containing SNP versus SFPs with multiple NP in order to test the biasness of detection, if any. Given the numbers of SFPs as verified by sequencing we observed that SFPs with SNP were detected as efficiently as SFPs with multiple NP (Table 4), an observation contrary to an earlier report [8].

**Table 4:** Nature of polymorphisms and detection sensitivity of SFPs.

(a) CP and LG			
Parameters	SFPs		
	1NP		Multiple-NP
	(1–5)	(6–20)	
SFP detected by gene-chip	8(10)	21(27)	48(63)
SFP escaped by gene-chip	9(33)	8(30)	10(37)
**Detection sensitivity (%)**	63		83
**(b) CP and RT**			
**Parameters**	**SFPs**		
	**1NP**		**Multiple-NP**
	(1–5)	(6–20)	
SFP detected by gene-chip	21(12)	87(48)	72(40)
SFP escaped by gene-chip	18(28)	18(28)	29(44)
**Detection sensitivity (%)**	75		71

Values in parentheses indicate percentage of SFP detected/escaped by gene-chip. Table includes SFP data analyzed at estimated 9.5% FDR in CP&LG and 9% FDR in CP&RT dataset.

CP = Cypress, LG = LaGrue, RT = RT0034, NP = nucleotide polymorphism.

### SFP comparison

The objective of present study was to find SFPs in CP&LG and CP&RT datasets as varieties in these two pairs constitute parents of two different mapping populations created to map QTLs/genes associated with milling yields trait [10]. The CP and LG belong to japonica [9] and RT to indica [10] background respectively. However with the available results in the three gene-chip predicted SFPs datasets we estimated frequency of overlapping SFPs. As expected we found significantly higher number of common SFPs between two japonica&indica datasets combinations viz. CP&RT and LG&RT (Table 5b). The above finding indicated not only the occurrence of common variations in the two japonica varieties (CP&LG) against indica variety (RT) but also efficacy of gene-chip method for predicting SFPs in the present study. Although common SFPs were also observed between diverse CP&LG and CP&RT (Table 5a) and also between CP&LG and LG&RT (Table 5c) datasets combinations respectively and expectedly the number of overlapping SFPs were much less. The chi-square test for independence for all three datasets combinations (Table 5) were highly significant showing strong association among the three pairwise comparison

**Table 5:** Overlapping SFPs among gene-chip experiments datasets.

(a) CP&LG vs. CP&RT					(b) CP&RT vs. LG&RT					(c) LG&RT vs. CP&LG				
		CP&RT					LG&RT					CP&LG		
		−SFP	non SFP	+SFP			−SFP	non SFP	+SFP			−SFP	non SFP	+SFP
CP&LG		45023	567496	15852	CP&RT		46718	561252	20581	LG&RT		6124	619458	2969
**−SFP**	**6124**	3482	2579	63	**−SFP**	**45203**	38031	6985	187	**−SFP**	**46718**	204	44915	1599
**non-SFP**	**619458**	41622	563339	14497	**non-SFP**	**567496**	8585	549761	9150	**non-SFP**	**561252**	3488	556461	1303
**+SFP**	**2969**	99	1578	1292	**+SFP**	**15852**	102	4506	11244	**+SFP**	**20581**	2432	18082	67
Chi-square = 43235.7, df = 4, p-value<2.2e-16					Chi-square = 35278.8, df = 4, p-value<2.2e-16					Chi-square = 650884.7, df = 4, p-value<2.2e-16				

The SFPs data have been taken from estimated FDR (delta) of 11.9% (delta = 2) in CP&LG, 10% (delta = 2) in CP&RT and 10% (delta = 1.6) in LG&RT respectively. The sign (+/−) indicates direction of polymorphism as explained in Figure-3 legend.

CP = Cypress, LG = LaGrue and RT = RT0034.

The availability of genome sequence information of Nipponbare [17] and 93-11 [18] allowed us to predict in-silico SFP candidates between above genetically diverged japonica and indica sub-species of rice respectively. Among the three gene-chips predicted SFPs datasets in the present study, the CP&RT and LG&RT pairs belongs to japonica and indica sub-species combination. The in-silico or computationally predicted SFPs in Nipponbare&93-11 (supplementary Table S3) were compared with gene-chip predicted SFPs, particularly with respect to japonica&indica datasets, in order to estimate overlapping SFPs among them. The significantly higher and comparable number of common polymorphism in two different japonica&indica datasets combination (Table 6b&c) reconfirms our earlier observation of occurrence of common variation between japonica&indica subspecies of rice (Table 5b). In gene-chip predicted SFPs comparison study in japonica&indica datasets combination ∼>70% of the total polymorphic SFP were common (Table 5b). This contrast with ∼30% of common SFPs observed in gene-chip vs. in-silico studies in two different japonica&indica datasets combination (Table 6b&c). The above discrepancy could be because (a) both japonica & indica varieties were different (b) in-silico predicted SFPs has significantly less number of +SFP compared to −SFP (supplementary Table S3) possibly because of the criteria used to predict +SFP (see methods; In silico SFP analyses) and (c) only 384998 features shared by in-silico and gene-chip were considered instead of ∼630000 features considered in only gene-chip predicted SFP comparison study. The higher number of common SFPs in gene-chip predicted SFPs of japonica&indica datasets combination (Table 5b) may be due to indica variety (RT) was common in both the datasets and secondly genetic divergence between two japonica varieties viz. CP and LG were low as evidenced by their frequency of polymorphisms (Table 2). From common polymorphisms data of gene-chip vs. in-silico SFPs comparison study of japonica&indica datasets combinations (Table 6b&c), we estimated further the frequency of overlapping SFPs by comparing their common SFPs. The analyses showed that 80–85% of SFPs were still common (Table 6d) in the three different japonica&indica datasets and also there wasn't any overlap with regards to polymorphism directions. This finding also supports the view that irrespective of varieties and the methods (gene-chip or in-silico) adopted for predicting SFPs, there will always be some common variation between japonica&indica varieties as evidenced by significantly higher number of common SFPs when two independently analyzed common polymorphism (Table 6b&c) were compared (Table 6d).

**Table 6:** Overlapping SFPs between datasets of gene-chip experiments and in-silico analyses.

(a) CP&LG vs. Nip&93-11					(b) CP&RT vs. Nip&93-11				
		Nip&93-11(in-silico)					Nip&93-11(in-silico)		
		−SFP	non-SFP	+SFP			−SFP	non-SFP	+SFP
CP&LG(gene-chip)		64774	316896	3328	CP&RT(gene-chip)		64774	316896	3328
**−SFP**	**3615**	1638	1908	69	**−SFP**	**28296**	17041	11113	142
**non-SFP**	**379530**	62277	314077	3176	**non-SFP**	**347932**	46144	299323	2465
**+SFP**	**1853**	859	911	83	**+SFP**	**8770**	1589	6460	721
Chi-sq. = 3711.629, df = 4, p-value<2.2e-16					Chi-sq. = 46973.71, df = 4, p-value<2.2e-16				
(c) LG&RT vs. Nip&93-11					(d) Common of common SFPs of (b) & (c)				
		Nip&93-11(in-silico)					(c)		
		−SFP	non-SFP	+SFP			−SFP		+SFP
LG&RT(gene-chip)		64774	316896	3328	(b)		17699		844
**−SFP**	**29369**	17669	11545	155	**−SFP**	**17401**	14817		0
**non-SFP**	**343875**	45012	296534	2329	**+SFP**	**721**	0		627
**+SFP**	**11754**	2093	8817	844					
Chi-sq. = 48561.51, df = 4, p-value<2.2e-16									

The gene-chip predicted SFPs data for CP&LG, CP&RT and LG&RT have been taken from estimated FDR (delta) of 11.9% (delta = 2), 10% (delta = 2)and 10% (delta = 1.6) respectively. The SFP data for Nip&93-11 have been taken from in-silico analyses(see methods; supplementary Table S3). The table has been generated from 384998 features shared by gene-chip and in-silico analyses. The sign(+/−) indicates direction of polymorphism as explained in Figure-3 and Table S3 legend. CP = Cypress, LG = LaGrue, RT = RT0034 and Nip = Nipponbare.

**Figure 4:**
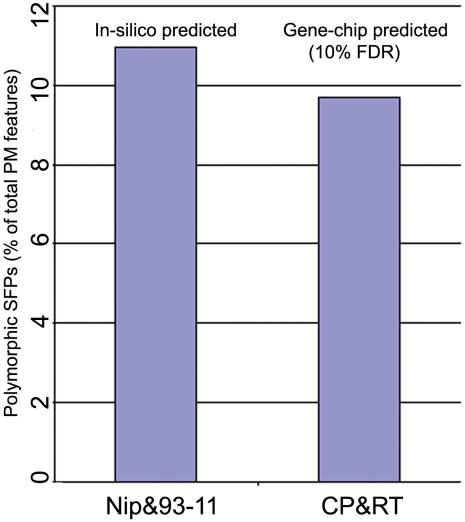
Polymorphic features predicted by in-silico analyses and gene-chip experiments. NIP = Nipponbare; CP = Cypress; RT = RT0034 and PM = perfect match.

All the computationally predicted SFPs (supplementary Table S3), irrespective of number associated with polymorphism direction (+/−), were considered as an example of approximate number of SFPs that can be predicted when the methods presented in the paper is used to derive SFP call between japonica and indica sub-species. Since SFPs in CP&RT pair was validated by sequence information, we compared the numbers of gene-chip predicted SFPs to those predicted by in-silico analyses. From gene-chip method at 9% estimated FDR, 4% of the total perfect match (PM) features present on the array (see methods) were polymorphic in CP&RT compared to 10.8% predicted by in-silico analyses in Nipponbare & 93-11 (Figure 4). However by lowering the stringency of estimated FDR to 10% the number of predicted SFPs in CP&RT dataset nearly doubled to 61055 that account 9.6% of the total PM features (Table 1b, Figure 4). Although validation of SFPs predicted at 10% estimated FDR increased the sensitivity of SFP detection considerably but simultaneously the observed FDR determined after sequencing also doubled to 20% compared to the estimated value by permutation (Table 3b). Considering the above results if we exclude ∼20% of the total SFP predicted at 10% estimated FDR in CP&RT dataset still ∼8% of total features present on the array are polymorphic compared to 10.8% predicted by in-silico analyses in Nipponbare & 93-11. The discrepancy in number of polymorphic features between the CP&RT and Nipponbare&93-11 datasets may be because of expected inherent diversity in their genome.

## Discussion

The phenotypic variations associated within organisms are products of underlying DNA diversity. Such variations in the nucleotides are great resources for development of molecular markers for mapping genes associated with either qualitative or quantitative traits. The genome wide polymorphism discovery captured as single feature polymorphism (SFP) resulting from differential hybridization of probes is a unique high-throughput approach for both genotyping and polymorphism discovery in a single assay [19]. Such strategy was highly successful in identifying polymorphism in yeast [5] and Arabidopsis [6] when probes generated from labeling of g-DNA of two varieties were hybridized to high density oligos expression arrays.

In the present study we tested the feasibility of such gene-chip based approach for polymorphism discovery in rice by hybridizing probes, generated from labeling of g-DNA, to rice whole genome expression array (Affymetrix). From verification of sequence information of predicted SFP conducted in two independent datasets viz. CP&RT and CP&LG, we found that SFPs can reliably be predicted in rice with ∼75% detection sensitivity (Table 3). Such a high rate of sensitivity is comparable to those reported in Arabidopsis [6], [20] but certainly more than barley [7] when similar DNA based method was used to predict SFP. The rice genome (389 Mb) is three times bigger than Arabidopsis (125 Mb) but much smaller than barley (5200 Mb) in size and comparatively higher efficiency of SFP detection in rice may be due greater representation of gene regions in probes as genome is less complex in size than barley. Although we observed considerable increase in detection sensitivity of SFPs at less stringent estimated FDR (Table 3), it was also accompanied by a significant increase in the observed FDR determined after sequencing compared to the estimated value by permutation. Since we couldn't find SFPs with higher d-stat values as false positive in both the datasets and the d-stat value of most of the false positive was close to the permuted cut-off level (data not shown), one can increase the likelihood of getting true SFPs *a priori* of their sequence analyses by deselecting SFPs having d-stat value close to permuted cut-off. Although a caution must be exercised in such approach as one may loose a considerable number of true SFPs also.

The rice varieties CP and LG are in japonica (tropical) [9] while RT in indica [10] genetic background respectively. The genetic differences between tropical and temperate japonica are very small [16] as compared to high degree of polymorphism observed between indica and japonica sub-species in rice [3]–[4], [15]. The gene-chip prediction of ∼6-7xs polymorphism in CP&RT or LG&RT compared to CP&LG dataset (Table 2) therefore mirrors the fact that varieties with similar genetic background are less polymorphic than to diverse genetic backgrounds. Additionally the pairs plot study (Figure 2) also supports the above observation as the number of features showing differential hybridization intensity was significantly more in CP&RT or LG&RT than in CP&LG dataset. The sensitivity of SFP discovery is further evidenced by the observation that SFP with SNP was detected as efficiently as SFP with multiple NP (Table 4); an observation in contrast to similar studies in barley [8]. Although variations ranging from single to multiple nucleotides were captured with nearly similar efficiency, SFP with SNP at flanking 1–5 bases of 25mer feature was detected poorly than those present in the middle (Table 4), a phenomenon similar to those reported earlier [7]. The interesting observation of significant number of common SFPs/polymorphism found among different japonica&indica datasets comparison studies (Table 5b and Table 6b,c&d) supports not only effectiveness of gene-chip approach for genome-wide polymorphism discovery but also provides a useful information regarding natural occurrence of common variations between japonica & indica subspecies.

Based on predicted SFPs, traditionally one can generate molecular markers once the fragment flanking SFP of interest is amplified and sequenced. Such type of approach can be useful to further narrow down the genetic interval of already identified QTLs. However given their higher sensitivity of detection (Table 3) together with dense coverage on genome, the SFPs data in rice can be used directly as molecular markers thus obviating the cumbersome process of marker development. With the defined physical location of SFPs on the chromosomes and the ease of generation of high density SFP data as demonstrated in the present study; their direct application as “molecular markers” will help substantially to constrain the genetic intervals containing “favorite genes” to high resolution thus making the prediction of candidate genes feasible provided genes present in the regions are annotated. The direct use of SFPs as “molecular markers” have been demonstrated in mapping genes associated with either qualitative [6] or quantitative traits [20]–[22] in Arabidopsis. In such QTL studies the probes generated from pooled DNA of RILs (recombinant inbred lines) showing extreme of phenotypes were used for hybridization and prediction of SFPs. Based on allelic frequency differences in both extreme pools, QTLs containing candidate genes were mapped with high resolution by extreme array mapping (XAM) in above studies.

Many of the plant traits of economic importance are generally controlled by numerous loci and to fine map genes associated with such traits is not trivial in terms of both time and resources. An alternative approach to speed up the process of gene discovery associated with quantitative traits that complements map-based cloning is association studies where candidate gene diversity is evaluated across natural populations and polymorphisms that correlate with phenotypic variation are identified [23]. The application of such approaches has been well demonstrated in humans by using gene-chip based SNP panels [24]. Since generation of SNP panels require prior sequence information and are quite expensive; the ease of generation of high density polymorphism (SFP) resulting from hybridization of probes to publicly available inexpensive expression array together with similar detection efficiency of SFP having either SNP or multiple NPs has promises that such SFPs data of rice can equally be used for associating functional variations with phenotypes similar to those suggested in Arabidopsis [25].

## Materials and methods

### Plant materials

The three rice varieties used in this study include Cypress, LaGrue and RT0034; first two belong to japonica while later to indica subspecies respectively. These rice varieties are the parents of two mapping population developed by RiceCAP [10] to map QTLs/genes associated with milling yield traits. The RT0034 & Cypress are parents of milling yield 1 (MY1) and Cypress & LaGrue of milling yield 2 (MY2) populations.

### Rice genome array

The rice genome array (www.affymetrix.com), designed for gene expression analyses, and contains probes to query 49,824 transcripts representing two rice cultivars with 48,564 from japonica and 1,260 from indica cultivar. The array is believed to represent about 46,000 distinct rice genes however the probesets from japonica is 54,168 and 1347 from indica subspecies. The arrays were designed using NCBI UniGene Build #52, (May7, 2004) incorporating predicted genes from GenBank® and the TIGR Os1 v2 data set (Affymetrix). Each probeset is represented by 11 perfect matches (PM) and an equal number of mismatch (MM) probes/features and each probe is 25 bp long. The array contains ∼630,000 PM probes/features from rice. The PM and MM probes positioned next to each other constitute a probe pair and each probe pair is distributed randomly but with defined position on the array or gene-chip.

### Probe generation and hybridization

DNA was isolated from leaves by CTAB method [26]. RNase treated DNA was phenol purified and dissolved in nuclease free water. The purified DNA was subjected to generation of biotin (biotin-dCTP) labeled probes by bio-prime labeling kit (Invitrogen). The reaction condition and parameters were same as described in Borevitz's methods paper [12] except that 400ng of purified DNA was used as template per reaction. The reaction was allowed to proceed for 16 hours at 20°C after which the product was ethanol purified and dissolved in 100 µl of nuclease free water. The amplified products were approximately ∼80–100 bp in size as verified on 1% agarose gel. From each labeling reaction 28–30 µg of products were obtained as quantified by nano-drop methods using default parameter set for DNA measurements. For each sample approximately 28 µg of reaction products generated from single reaction were used for hybridization to Affymetrix rice expression array at DNA core facility (http://www.biotech.missouri.edu/dnacore/). The hybridization and washing was performed according to standard RNA protocol as described in Affymetrix manual.

### Data analyses

The data quality was assessed from expression report generated by GCOS1.3 software using the default parameter set for rice genome array. The raw intensity values of all the probes present on the chip was transferred from .cel files into .txt file by using the tools available on GCOS1.3 software. The intensity values of only PM (perfect match) probes/features (628,551) were subsequently extracted and subjected to data analyses as described [12]–[13]. Briefly, intensity data was preprocessed by RMA2 and quantile normalized using Affy package (www.bioconductor.org). The log2 transformed intensity value of each feature was subsequently used to derive SFP call by publicly available *siggene* package (www.bioconductor.org) and scripts and codes [12] for such statistical analyses.

### PCR and sequencing

DNA fragments of 200–300 bp flanking the SFP regions were amplified from genomic DNA. The sequence information of features/probes was obtained from Affymetrix (www.affymetrix.com). Primers were designed on the basis of known sequence information of Nipponbare genome (TIGRv3) by using the blast tool of *Gramene* (www.gramene.org). The thermal cycle program for PCR were 95°C for 2 min, 28 cycles of 95°C for 15 sec, 58°C for 15 sec, 72°C for 30 sec and with final extension at 72°C for 5 min. The PCR conditions were; 1× of 10× Ex Taq buffer (Mg^+^), 125 µM of each dNTPs, 0.5 units of DNA polymerase, 0.5 µM each primer, 1 µl of genomic DNA to a final volume of 40 µl. In order to get DNA fragments without any error during amplification, PCR was performed with hot-start Ex Taq DNA polymerase (Panvera US, Madision WI) having proof reading activity. PCR products showing single bands were purified (PCR purification kit, Qiagen) and subjected to sequencing by Applied Biosystems 3730 DNA Analyzer using Prism Big Dye Terminator cycle sequencing chemistry at DNA core facility.

### SFP confirmation

For sequence validation only those SFPs were considered whose corresponding probe/feature sequence had unique location and perfect match (PM) on Nipponbare genome (TIGRv3). In the present study any variation in sequence ranging from substitution or indel involving single (SNPs) to multiple bases were considered while determining the nature of polymorphism associated with SFPs. High quality single reads, as obtained from ABI chromatograms, were used for sequence comparison with publicly available vectorNTI tools (Invitrogen).

### In silico SFP analyses

The Affymetrix rice whole genome expression array has been designed mainly on the sequence from japonica sub-species cv. Nipponbare. The sequences of all ∼630,000 25mer PM probes/features sequences present on the array were compared (megablast and E-value: 1e-4) against Nipponbare genome build 4[17] to remove features that were repetitive and not perfect matches. From above analyses we identified 382205 features that were perfect match (100% identity, 25/25) and each feature was having unique location on genome (supplementary Table S3). When sequences of these unique features were compared (megablast and E-value: 1e-4) against 93-11 genome [18], altogether 64883 features showing changes ranging from single to multiple nucleotides and also no matches were identified and were considered together as in-silico predicted −SFP candidates (i.e. Nip>93-11) and rest as non-SFPs (Nip = 93-11). The sequences of remaining features (25mer) from above analyses that didn't show 100% identity on Nipponbare genome, were compared (megablast and E-value: 1e-4) against 93-11 genome and information of unique features showing 100% identity (25/25) and also single location on genome were extracted. When sequences of these unique features were compared (megablast and E-value: 1e-4) against Nipponbare genome, information of 3344 features showing not only variations in their sequences but also having unique location were extracted and were considered as in-silico predicted +SFP candidates (i.e. 93-11>Nip). In determining SFPs (+/−), we considered their unique position with reference to Nipponbare genome only.

## Supporting Information

Table S1 Sequence verified SFPs in CP& LG dataset. Changes in sequences are highlighted in blue and black; - indicates deletion; CP = Cypress and LG = LaGrue.
(0.03 MB XLS)

Table S2 Sequence verified SFPs in CP&RT dataset. Changes in sequences are highlighted in blue and black; - indicates deletion; CP = Cypress and RT = RT0034.
(0.05 MB XLS)

Table S3 In-silico predicted SFPs and their distribution. Nip = Nipponbare, −SFP(Nip>93-11), +SFP (93-11>Nip), non SFP (Nip = 93-11)
(0.02 MB XLS)
